# Validation of a Markerless Non-Contact Gait Analysis System for Three-Dimensional Gait Kinematics in Patients with Ankle Injuries: A Concurrent Comparison Study with the Vicon Three-Dimensional Motion Capture System

**DOI:** 10.3390/s26144579

**Published:** 2026-07-19

**Authors:** Xuhan Cao, Qing Zhou, Shuo Wang, Mingjie Dong, Shuang Ren, Qinwei Guo

**Affiliations:** 1 Department of Sports Medicine, Peking University Third Hospital, Institute of Sports Medicine of Peking University, Beijing 100191, China; caoxuhan0712@sina.com (X.C.); zhouqingdr@163.com (Q.Z.); 2 Beijing Key Laboratory of Research and Translation for Drugs and Medical Devices in Precision Diagnosis and Treatment of Sports Injuries, Beijing 100191, China; 3 Engineering Research Center of Sports Trauma Treatment Technology and Devices, Ministry of Education, Beijing 100191, China; 4 Department of Engineering Physics, Key Laboratory of Particle and Radiation Imaging, Ministry of Education, Tsinghua University, Beijing 100084, China; shuo-wan19@tsinghua.org.cn; 5 College of Mechanical & Energy Engineering, Beijing University of Technology, Beijing 100124, China; dongmj@bjut.edu.cn

**Keywords:** gait analysis, markerless motion capture, vision transformer, validation, ankle injury

## Abstract

**Highlights:**

**What are the main findings?**

- Foot4D demonstrated good-to-excellent agreement with Vicon for all 12 spatiotemporal gait parameters (ICC = 0.738–0.999) in patients with ankle injuries, with gait cycle time showing near-perfect concordance (ICC = 0.999).
- Lower limb 3D kinematic agreement followed a proximal-to-distal gradient (hip ICC ≈ 0.947 > knee ICC ≈ 0.921 > ankle ICC ≈ 0.839), with the ankle frontal and transverse planes at the lower bound of good agreement.

**What are the implications of the main findings?**

- Foot4D provides a viable markerless clinical alternative for routine spatiotemporal and proximal joint gait assessment in ankle injury patients.
- The reduced accuracy of ankle multiplanar kinematics highlights the need for targeted algorithmic refinements to advance markerless motion capture for precise foot–ankle evaluation.

**Abstract:**

(1) Background: This study validated the accuracy of a markerless gait analysis system, Foot4D, against the Vicon 3D motion capture system in 60 patients with ankle injuries. (2) Methods: Synchronized gait data were collected using both systems while participants walked on a treadmill at self-selected speeds. The Foot4D system employs a Vision Transformer (ViT-H/16) encoder and the SKEL parametric model for markerless 3D pose reconstruction from multi-view depth cameras. Spatiotemporal gait parameters and lower limb 3D kinematics (hip, knee, and ankle) were compared using ICC, Bland–Altman analysis, and MAE. (3) Results: All 12 spatiotemporal parameters showed good-to-excellent agreement (ICC = 0.738–0.999, MAE = 0.009–0.108 m/s). Agreement for joint kinematics decreased from proximal to distal: hip (mean ICC ≈ 0.947, MAE 1.16–1.52°), knee (mean ICC ≈ 0.921, MAE 1.17–1.75°), and ankle (mean ICC ≈ 0.839). Ankle sagittal plane ROM demonstrated good agreement (ICC = 0.857–0.911), while the frontal (ICC ≈ 0.807) and transverse (ICC ≈ 0.816–0.835) planes were at the lower bound of good agreement, with a −3.19° systematic bias in the injured ankle sagittal plane. (4) Conclusions: Foot4D is clinically reliable for spatiotemporal and proximal joint kinematic assessment, though ankle multiplanar motion measurement warrants further algorithmic optimization.

## 1. Introduction

Gait is a periodic locomotor pattern characterized by individual specificity that emerges during human walking. As a complex physiological process integrating neuromuscular control, muscular synergy, and biomechanical mechanisms, gait is modulated by multiple intrinsic and extrinsic factors [1,2,3,4], and its execution critically depends on the structural integrity and functional coordination of the lower limb joints. The ankle serves as a pivotal component in the gait cycle, bearing load and generating propulsion. Its motion is characterized by small amplitude, rapid changes, and complex three-dimensional coupling, exerting a decisive influence on overall gait stability, propulsive efficiency, and energy metabolism. Studies have demonstrated that ankle dysfunction and injury induce characteristic alterations in walking patterns, including increased gait asymmetry, compromised dynamic stability, and compensatory strategies at proximal joints such as the knee and hip [5,6,7]. Therefore, high-precision, high-resolution measurement of lower limb three-dimensional kinematics and spatiotemporal gait parameters is essential for objectively quantifying ankle functional limitation, understanding injury mechanisms, and guiding precision rehabilitation. However, existing validation studies of markerless motion capture systems have predominantly focused on healthy populations; their accuracy under pathological gait conditions accompanied by joint motion restriction, gait asymmetry, and compensatory movements remains unclear. Selecting patients with ankle injury as the validation cohort not only tests the tracking robustness of the system under clinically realistic complex motions but also enables a comprehensive evaluation of the technical boundaries of the system in measuring multi-joint three-dimensional kinematics of the lower limb through asymmetric patterns between the affected and healthy Sides and proximal joint compensatory movements.

Gait analysis serves as a critical tool for evaluating motor function, diagnosing neuromusculoskeletal disorders, and monitoring rehabilitation progress, with its methodology having evolved from subjective observation to objective quantification [8]. Traditional clinical assessment relies on visual observation by therapists, yet suffers from limitations including low reliability, dependence on experience, and an inability to quantify subtle abnormalities [9]. To obtain precise and reproducible parameters, instrumented gait analysis techniques have emerged, with devices such as optical motion capture systems [10,11,12,13] enabling comprehensive quantification of joint kinematics and related metrics. Three-dimensional optoelectronic motion capture systems based on retroreflective markers, such as Vicon (Vicon Motion Systems, Oxford, UK) and Qualisys, are regarded as the “gold standard” [14,15,16], serving as the reference benchmark owing to their high accuracy and reliability. Nevertheless, these systems are costly, require dedicated laboratory environments, involve complex operation, and require the attachment of markers, which may introduce skin artifacts and interfere with natural gait, thereby limiting their widespread clinical adoption. To overcome these limitations, markerless gait analysis technologies based on computer vision and deep learning pose estimation algorithms have been developed. These systems automatically parse joint kinematic data from video streams without the need for surface markers, offering advantages such as minimal subject preparation, non-invasiveness, and applicability outside laboratory settings [17], thereby significantly enhancing clinical accessibility. Early depth-sensing devices such as Kinect, although portable and low-cost, exhibited discrepancies in accuracy and reliability compared with the gold standard [12,18,19,20]. In recent years, two-dimensional/three-dimensional human pose estimation frameworks such as OpenPose [21] and MediaPipe [8] have achieved substantial breakthroughs, enabling real-time keypoint detection via conventional cameras. With the advancement of deep learning architectures, Transformer architectures centered on self-attention mechanisms have demonstrated remarkable advantages in modeling global contextual information [22,23,24], providing novel pathways for three-dimensional pose estimation in complex dynamic scenarios.

However, existing systems have predominantly emphasized whole-body or multi-joint global analysis [17,25], with their algorithmic models and camera configurations not specifically optimized for capturing fine ankle motion. Consequently, they exhibit insufficient accuracy or noise sensitivity when measuring the complex three-dimensional motion of the ankle, particularly in the frontal and transverse planes. To address this need, the present study validates the markerless gait analysis system Foot4D, which is built upon a Vision Transformer (ViT-H/16) image encoder and Transformer decoder architecture. The system directly regresses parameterized human model parameters from a multi-view depth camera array (Azure Kinect DK and Intel RealSense D415, hardware-synchronized at 30 fps). It employs the SKEL (Skeletal Kinematics Enveloped by a Learned body model) model [26], which integrates high-precision biomechanical skeletal data through skeletal regression techniques built upon the standard SMPL (Skinned Multi-Person Linear model), achieving precise mapping from surface morphology to the anatomical skeleton and notably improving the modeling capability for complex foot and ankle motion. Technically, Foot4D differentiates itself from existing markerless frameworks in four key dimensions. First, unlike the CNN-based encoders employed in OpenPose [21] and MediaPipe [8], the ViT-H/16 architecture leverages self-attention mechanisms to model long-range spatial dependencies, facilitating more robust visual feature extraction in anatomically complex regions such as the foot–ankle. Second, while conventional frameworks output surface keypoints or standard SMPL parameters, the SKEL model incorporates an anatomical regression matrix trained on registered MRI/CT datasets, enabling direct derivation of deep skeletal landmarks through soft tissue penetration—a capability critical for accurate joint center localization in the foot–ankle region. Third, the multi-view depth camera array (six synchronized units) mitigates the depth ambiguity and occlusion limitations inherent to monocular or dual-camera setups. Fourth, treadmill-anchored in situ self-calibration eliminates manual wand calibration, enhancing clinical workflow efficiency. The present study seeks to determine whether this integrated architecture overcomes the accuracy limitations that have constrained existing markerless systems in fine-scale foot–ankle kinematic measurement.

This study employs the Vicon three-dimensional optoelectronic motion capture system as the concurrent reference standard, synchronously acquiring gait data in patients with ankle injury to evaluate the accuracy and inter-system agreement of the Foot4D system in measuring lower limb three-dimensional kinematics and spatiotemporal gait parameters, thereby providing methodological validation for the application of markerless optical motion capture technology in fine-scale joint kinematic measurement [27].

## 2. Materials and Methods

### 2.1. Participants

Previous internal validity studies in healthy populations have indicated that a minimum sample size of 20 participants is typically required to obtain reliable results [16,28,29]. In this study, a total of 60 patients with ankle injury who were scheduled for surgical treatment at the Department of Sports Medicine of our hospital between January 2025 and October 2025 were enrolled as participants. The cohort comprised 32 males and 28 females, with an age range of 18–65 years (mean, 33 ± 10.3 years), a mean height of 168.1 ± 6.1 cm, and a mean body mass of 63.7 ± 10.3 kg. All enrolled participants were screened to exclude joint fibrosis, joint ankylosis, and significant motion limitation. During testing, participants walked barefoot on the treadmill to eliminate footwear-induced kinematic variability and ensure optimal visibility of foot–ankle anatomy for the markerless system. Participants wore standardized tight-fitting shorts to eliminate clothing interference with marker tracking. This study was conducted in accordance with the principles of the Declaration of Helsinki. The study protocol was reviewed and approved by the Ethics Committee of Peking University Third Hospital (approval number: IRB00006761-M2024734), and all participants provided written informed consent prior to participation.

### 2.2. Instrumentation

**Figure 1:**
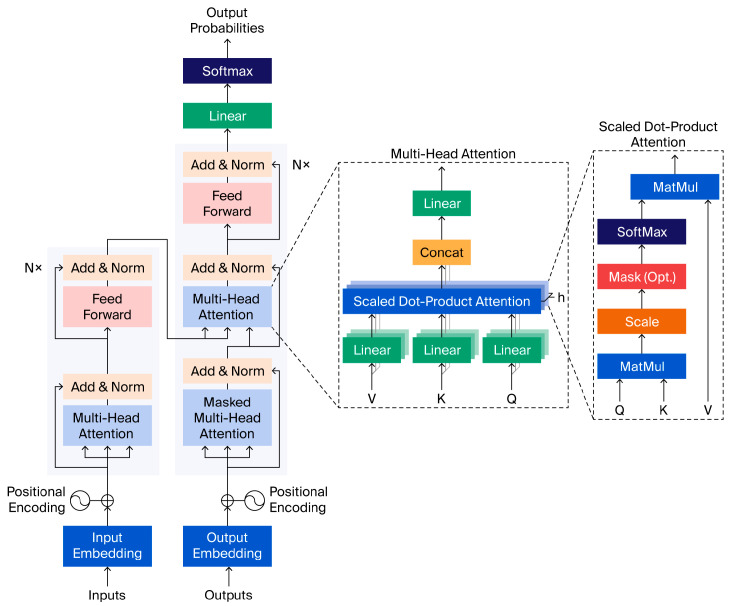
Transformer network architecture of the Foot4D system. The pre-trained ViT-H/16 encoder extracts features from binocular video (60 fps, 1920 × 1080 pixels), and a six-layer Transformer decoder completes three-dimensional pose reconstruction. The SKEL model, based on SMPL skeletal regression, improves joint position accuracy and kinematic interpretability, enhancing the modeling capability for complex foot and ankle motion.

**Figure 2:**
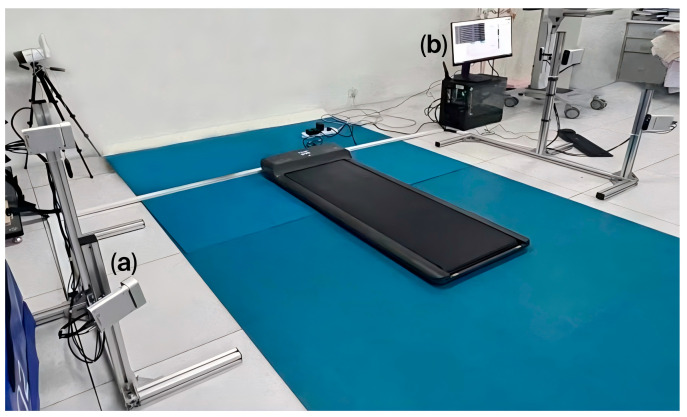
Foot4D gait testing equipment. (**a**) Multi-view depth camera array and treadmill system; (**b**) acquisition host and force platform interface.

The Foot4D system employed binocular cameras (60 fps, 1920 × 1080 pixels) to achieve markerless three-dimensional pose reconstruction via a pre-trained ViT-H/16 encoder and a six-layer Transformer decoder (Figure 1). The binocular RGB cameras operated at 60 fps and served as the primary visual input for the ViT-H/16 image encoder. Separately, kinematic data acquisition was performed by a depth camera array consisting of four Azure Kinect DK (time-of-flight) and two Intel RealSense D415 (binocular stereo vision) units, hardware-synchronized at 30 fps (Figure 2). Temporal alignment between the 60 fps RGB stream and the 30 fps depth stream is achieved via a frame-level nearest-neighbor strategy. For each RGB frame, the depth point cloud from the temporally closest hardware-synchronized depth frame was retrieved (maximum temporal offset ≤ 8.33 ms). This frame fusion produced a unified 60 Hz data stream that drove the kinematic reconstruction pipeline. Multi-view point cloud fusion was employed to generate global dynamic sequences and mitigate occlusion effects.

**Figure 3:**
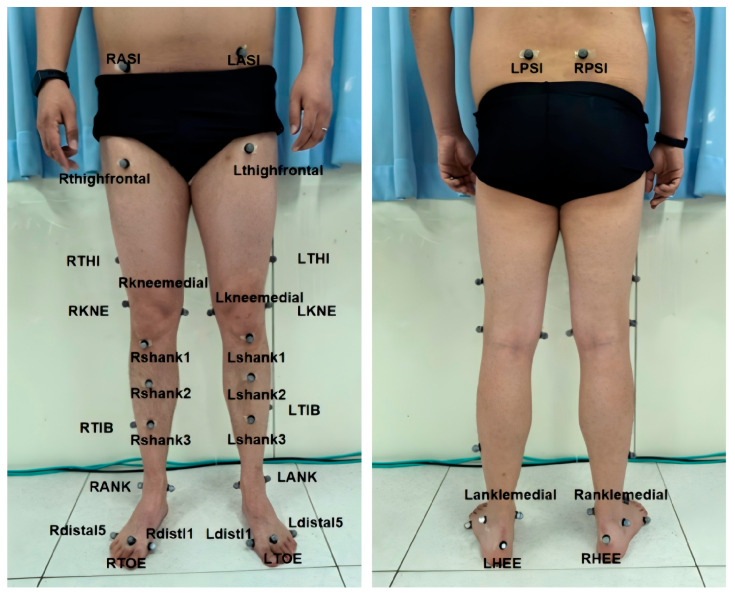
Vicon anatomical retroreflective spherical markers. Left/right anterior superior iliac spines (LASI/RASI), left/right posterior superior iliac spines (LPSI/RPSI), left/right anterior thighs (Lthighfrontal/Rthighfrontal), left/right lateral upper one-third of the thighs (LTHI/RTHI), left/right medial knees (Lkneemedial/Rkneemedial), left/right lateral femoral condyles (LKNE/RKNE), left/right shank zone 1 (Lshank1/Rshank1), left/right shank zone 2 (Lshank2/Rshank2), left/right shank zone 3 (Lshank3/Rshank3), left/right lateral lower one-third of the shanks (LTIB/RTIB), left/right lateral malleoli (LANK/RANK), left/right medial ankles (Lanklemedial/Ranklemedial), left/right plantar/toes (LPLANK/RPLANK), left/right toes (LTOE/RTOE), left/right distal fifth toes (Ldistal5/Rdistal5), and left/right distal first toes (Ldistl1/Rdistl1).

In this study, the Vicon motion analysis system (Vicon Motion Systems, Oxford, UK) was employed for three-dimensional motion capture. The system consisted of eight T10 infrared cameras and Vicon Nexus software (version 1.8.5), with a sampling frequency of 100 Hz. Given that this study focused primarily on lower limb kinematics, marker placement was concentrated on lower limb anatomical landmarks. The marker placement protocol followed the Vicon Plug-in Gait lower limb model (Vicon Motion Systems, Oxford, UK), a standardized marker configuration widely adopted in clinical gait analysis. Prior to testing, 32 retroreflective markers were attached to the body surface of each participant according to this protocol, including the bilateral posterior superior iliac spines, anterior superior iliac spines, greater trochanters, anterior thighs, medial/lateral femoral epicondyles, tibial tuberosities, anterior shanks, medial/lateral malleoli, first and fifth metatarsophalangeal joints, the midpoint of the second-to-third metatarsophalangeal joints, and the calcanei. Static calibration data were also collected (Figure 3).

### 2.3. Data Acquisition and Processing

**Temporal pipeline summary:** The Foot4D system produces kinematic data through a four-stage temporal pipeline: (1) dual-stream acquisition (RGB at 60 fps and depth at 30 fps); (2) frame-level temporal nearest-neighbor alignment, yielding a fused 60 Hz stream; (3) ViT-SKEL pose reconstruction, outputting kinematic parameters at 60 Hz; and (4) linear interpolation resampling from 60 Hz to 100 Hz for inter-system synchronization with Vicon. The mean synchronization error across all participants was 4.2 ± 2.8 ms, confirming sub-frame temporal alignment accuracy.

**Inter-system synchronization:** Synchronization between the two systems was achieved via an event synchronization method. Prior to data collection, participants stood still on the treadmill, and the researcher struck the edge of the force platform with a wooden mallet to generate a vibration signal. This signal was simultaneously recorded by both systems and served as the temporal zero point for aligning subsequent data. To unify the sampling rate, Foot4D data (60 Hz) were resampled to match the Vicon system (100 Hz) using linear interpolation. Linear interpolation was chosen because the resampling ratio was modest (60 Hz to 100 Hz; a factor of 1.67), and previous studies have demonstrated that linear interpolation introduces minimal phase distortion for such frequency ratios while preserving the temporal characteristics of gait data [30]. Higher-order interpolation methods (e.g., cubic spline) were not deemed necessary as they do not substantially improve accuracy for this resampling ratio and may introduce edge artifacts. The synchronization error was quantified by computing the temporal difference between the vibration peaks detected in both systems. The mean synchronization error across all 60 participants was 4.2 +/− 2.8 ms, with a maximum discrepancy of 9.1 ms, confirming that the inter-system synchronization error remained below 10 ms.

**Calibration and static acquisition:** Following synchronization, system calibration and static acquisition were performed. Participants stood still at a predefined position to complete calibration, and the static skeletal model for the Vicon system was established.

**Figure 4:**
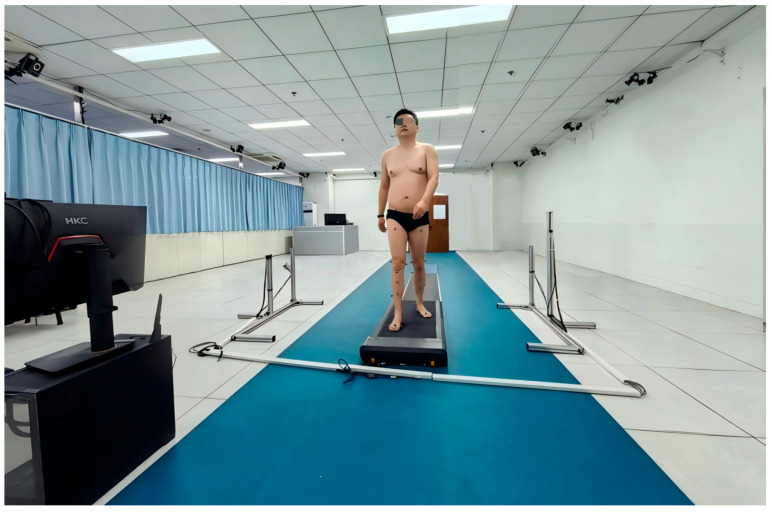
Gait testing scenario.

**Dynamic acquisition:** During dynamic acquisition, participants walked naturally on the treadmill at a self-selected comfortable speed (0.7–1.2 m/s). Each continuous acquisition lasted 30 s (approximately 20–25 gait cycles) and was repeated five times, with a 2 min rest interval between trials. The Vicon system continuously captured marker trajectories, while the Foot4D system simultaneously acquired markerless dynamic video via its cameras (Figure 4).

**Data extraction:** From the Vicon data, five consecutive gait cycles with good image quality and stable gait were selected. The Foot4D system extracted data from the corresponding time segments using the synchronization timestamp to ensure temporal matching. The same number of gait cycles was extracted from both systems for each participant. Five complete gait cycles were extracted per participant per trial, yielding a total of 25 cycles. For each participant, the mean value across all 25 extracted gait cycles (5 trials × 5 cycles) was computed for each parameter, and these participant-level means (n = 60) were used for the inter-system agreement analysis. This approach reduces intra-subject variability and provides a more stable estimate of each participant’s true gait characteristic.

### 2.4. In Situ Spatial Self-Calibration of the Multi-Camera System

**Figure 5:**
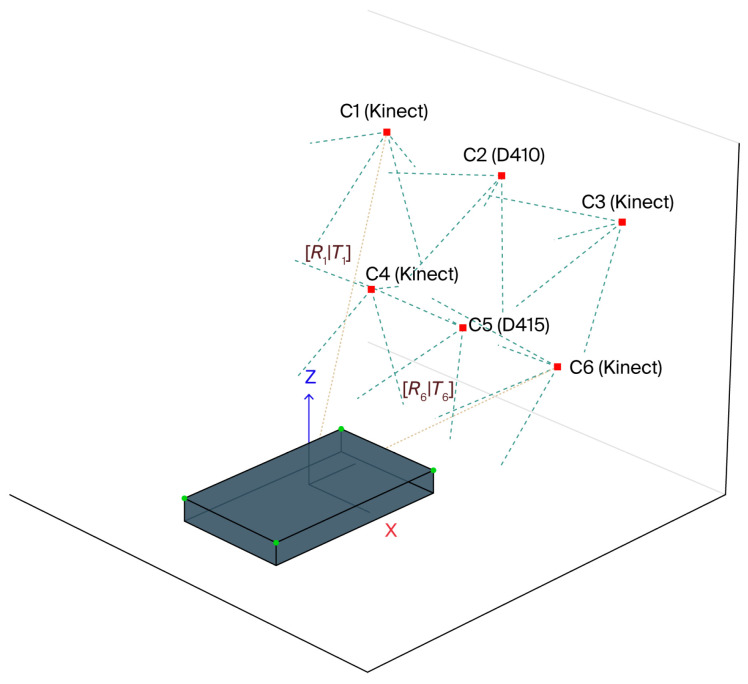
Schematic of the in situ spatial self-calibration principle for the Foot4D multi-camera system. The global world coordinate system (World Origin, 0, 0, 0) is anchored to the geometric center of the treadmill frame, with the X-, Y-, and Z-axes corresponding to the participant’s coronal, sagittal, and vertical axes, respectively. The viewing frusta of the six depth cameras (C1–C6) converge at the core capture region.The dashed lines extending from each camera indicate the viewing frusta of the six depth cameras (C1–C6), representing the three-dimensional field-of-view boundaries of each camera, which converge at the core capture region. The system automatically identifies frame feature points (green dots) and solves, in real time, the transformation matrices from each camera’s local coordinate system to the global coordinate system.

The Foot4D system adopted a two-stage strategy combining factory pre-calibration of intrinsic parameters with in situ self-calibration of extrinsic parameters. The origin of the global coordinate system was anchored to the geometric center of the treadmill frame, with the X-, Y-, and Z-axes corresponding to the coronal, sagittal, and vertical axes, respectively. The viewing frusta of the six depth cameras converged at the core capture region (Figure 5). This scene-prior-based calibration strategy eliminated the traditional manual wand-waving calibration step, ensuring spatial consistency for multimodal data fusion. Detailed mathematical formulations of the calibration procedure are provided in Appendix A.

### 2.5. SKEL-Based Markerless Kinematic Reconstruction Algorithm

The Foot4D system employed the SKEL parametric human body model for three-dimensional motion reconstruction. This model, based on SMPL, drives a three-dimensional mesh through shape parameters β and pose parameters θ, generating a surface model via linear blend skinning:

$$M\left(\beta ,\theta \right)=W\left(T\left(\beta ,\theta \right),J\left(\beta \right),\theta ,W\right)$$

where T denotes the template mesh, J denotes the joint centers, and W denotes the skinning weight matrix.

The system fitted the multi-view point cloud by minimizing the energy function:

$$E\left(\beta ,\theta \right)=\lambda_{data}E_{data}+\lambda_{prior}E_{prior}$$

where the data term *E_data_* constrains the model to fit the observed point cloud, and the prior term *E_prior_* introduces pose regularization and collision detection to prevent anomalous solutions. Following optimization, the anatomical regression matrix *R_anat_* mapped the surface model to anatomical landmarks conforming to the International Society of Biomechanics (ISB) standards:

$$J_{anat}=R_{anat}M\left(\beta^{*},\theta^{*}\right)$$

This regression matrix was trained on medical imaging data, enabling the derivation of three-dimensional coordinates of deep skeletal landmarks through soft tissue penetration, thereby achieving precise mapping from surface morphology to the anatomical skeleton. The complete algorithmic framework is detailed in Appendix B.

### 2.6. Three-Dimensional “Hip–Knee–Ankle” Joint Angle Calculation Model

**Figure 6:**
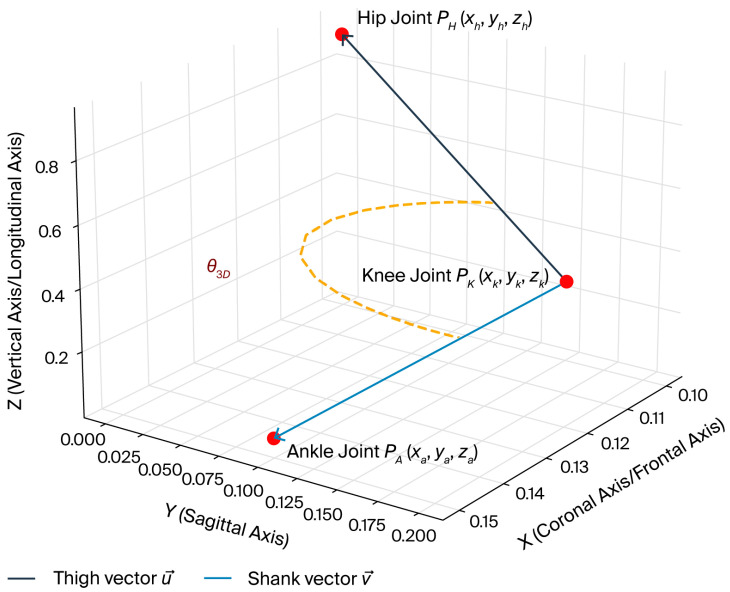
Three-dimensional spatial vector-based hip–knee–ankle joint angle calculation model. In the global coordinate system defined by the coronal axis (X), sagittal axis (Y), and vertical axis (Z), the SKEL model localizes the three-dimensional spatial coordinates of the hip joint $P_{H}$, knee joint $P_{K}$, and ankle joint $P_{A}$. Taking the knee joint $P_{K}$ as the rotation center, the thigh vector $\vec{u}$ and shank vector $\vec{v}$ define the three-dimensional absolute angle $\theta_{3D}$ (orange dashed arc), eliminating two-dimensional projection distortion and supporting precise decomposition of kinematic data into specific anatomical planes.

Based on the anatomical landmarks output by the SKEL model, a spatial vector model was constructed in the global coordinate system *O–XYZ* (Figure 6). Taking the knee joint as an example, let the hip, knee, and ankle joint centers be denoted as *P_H_*(*x_h_y_h_*,*z_h_*), *P_K_*(*x_k_*,*y_k_*,*z_k_*) and *P_A_*(*x_a_*,*y_a_*,*z_a_*), respectively. The thigh vector $\vec{u}$ and shank vector $\vec{v}$ are defined as:

$$\vec{u}=P_{H}-P_{K}=\left(x_{h}-x_{k},y_{h}-y_{k},z_{h}-z_{k}\right)$$

$$P_{A}-P_{K}=\left(x_{a}-x_{k},y_{a}-y_{k},z_{a}-z_{k}\right)$$

The complete derivation of anatomical plane projection is provided in Appendix C.

Three-dimensional absolute angle:

$$\theta_{3D}=\arccos\left(\frac{\vec{u}\cdot \vec{v}}{\left|\vec{u}\right|\left|\vec{v}\right|}\right)$$

Projection onto the sagittal plane (Y–Z plane) yields the clinical flexion–extension angle:

$$\theta_{flex}=180^{\circ }-\arccos\left(\frac{\vec{u}sag\cdot \vec{v}sag}{\left|\vec{u}sag\right|\left|\vec{v}sag\right|}\right)$$

It should be noted that the joint angles calculated in the present study were derived using a three-dimensional spatial vector projection approach based on SKEL-estimated anatomical landmarks, rather than a full segment-fixed local coordinate system and Cardan/Euler decomposition as recommended by the ISB. This approach projects anatomical segment vectors onto the global sagittal, coronal, and transverse planes to obtain clinically interpretable planar ROM measures. Therefore, the resulting values should be interpreted primarily as plane-specific vector projection ROMs rather than strict ISB Cardan angles.

This strategy was adopted because the primary aim of the present study was to evaluate concurrent agreement between Foot4D and Vicon using matched computational definitions, while minimizing instability associated with markerless estimation of complete segment-fixed coordinate systems. Nevertheless, we acknowledge that this method may not fully account for segmental torsion, individualized anatomical axes, or cross-talk caused by out-of-plane rotations. For applications requiring rigorous multiplanar joint kinematics, ISB-recommended local coordinate system-based Cardan analysis remains preferable.

### 2.7. Gait Event Identification and Spatiotemporal Parameter Calculation

#### 2.7.1. Data Processing and Gait Event Identification

As participants walked on a treadmill, the vertical displacement (*Z*-axis) of the foot was constrained by the treadmill belt, and force platform data were unavailable. Although the experimental setup included a force platform beneath the treadmill, the ground reaction force (GRF) signals acquired during treadmill walking were not suitable for reliable gait event identification due to four technical factors: (1) the force platform measures the total dynamic load of the treadmill frame, belt, motor, and participant combined, rather than isolated foot–ground contact forces; (2) continuous belt motion introduces persistent low-frequency vibration noise that obscures transient foot strike signals; (3) during double support, both feet simultaneously contact the integrated treadmill belt, preventing unambiguous contralateral strike timing identification; and (4) the measured force signal reflects treadmill belt dynamics (acceleration and tension variation) superimposed on foot contact forces, making threshold-based heel strike/toe-off detection unreliable. For these reasons, the force platform was employed exclusively for inter-system temporal synchronization (via a manually generated vibration impulse; see Section 2.3) but not for automated gait event detection. Therefore, gait events were identified using an “extrema method” based on the anterior–posterior (*Y*-axis) trajectory of the foot markers. Heel strike (HS) was defined as the local positive maximum of the heel marker along the *Y*-axis (the farthest forward point during the gait cycle), and toe-off (TO) was defined as the local negative minimum of the toe marker along the *Y*-axis (the farthest backward point as the treadmill belt moved). Prior to the extrema search, all three-dimensional marker trajectories were smoothed using a fourth-order zero-phase Butterworth low-pass filter (cutoff frequency, 6 Hz) to eliminate high-frequency soft tissue artifacts and instrument noise.

#### 2.7.2. Mathematical Calculation Model for Spatiotemporal Parameters

Following gait event extraction, spatiotemporal parameters were calculated based on event timestamps and marker coordinates. Let t_HS1_ and t_HS2_ denote two consecutive ipsilateral heel strikes, t_cHS_ denote the contralateral heel strike within that interval, and t_TO_ denote the ipsilateral toe-off. The three categories of parameters are defined as follows:

**Temporal parameters:** These included gait cycle time, stance phase duration, swing phase duration, double-support duration, single-support duration, and cadence (steps per minute). For example, gait cycle time ($T_{cyc}$) was calculated as the time difference between two consecutive ipsilateral heel strikes:

$$T_{cyc}=t_{HS2}-t_{HS1}$$

**Spatial parameters:** These included step length, stride length, step width, and foot progression angle. All spatial parameters were calculated as relative Euclidean distances between foot markers in the transverse plane (X-Y plane) to exclude the influence of treadmill belt motion. For example, step width (w) was defined as the absolute distance between the bilateral heel markers in the coronal plane (*X*-axis, i.e., the mediolateral direction) at heel strike:

$$w=\left|x_{R}\left(t_{HS1}\right)-x_{L}\left(t_{HS1}\right)\right|$$

**Velocity parameters:** The equivalent walking speed (v) was calculated as the ratio of stride length to gait cycle time:

$$v=\frac{L}{T_{cyc}}$$

(Complete mathematical formulations are provided in Appendix D).

### 2.8. Outcome Measures

The outcome measures comprised spatiotemporal parameters (gait cycle time, step time, stance phase duration, swing phase duration, double-support duration, single-support duration, cadence, step length, stride length, walking speed, step width, and foot progression angle) and joint motion parameters for the affected and healthy Sides (hip joint angle, knee joint angle, and ankle joint angle).

### 2.9. Statistical Analysis

All statistical analyses were performed using SPSS 25.0 (IBM Corp., Armonk, NY, USA). Continuous data are presented as mean +/− standard deviation. Prior to parametric testing, the normality of the data distribution was assessed using the Shapiro–Wilk test. All continuous variables met the normality assumption (*p* > 0.05), justifying the use of parametric tests. To evaluate the agreement between the Foot4D system and the Vicon system, the following methods were employed:

(1) Paired-samples *t*-test: To examine whether statistically significant differences existed between the mean values of the two datasets.

(2) Intraclass correlation coefficient (ICC): The ICC(2,1) two-way random-effects model with absolute agreement was selected to assess the consistency and reliability between the two systems. This model was chosen because both raters (measurement systems) and subjects were considered random samples from their respective populations, and we sought to evaluate the degree of absolute correspondence between the two systems rather than consistency alone. ICC values were interpreted as follows: <0.5 poor, 0.5–0.75 moderate, 0.75–0.9 good, and >0.9 excellent.

(3) Bland–Altman analysis: Bland–Altman plots were constructed, and the limits of agreement (LoA, defined as the mean difference ± 1.96 × standard deviation) were calculated to visually display the distribution of measurement differences between the two methods and identify potential systematic bias.

(4) Pearson correlation coefficient: To evaluate the linear correlation between the two datasets. Pearson’s correlation was employed because the data met the normality assumption (Shapiro–Wilk test, *p* > 0.05). Spearman’s rank correlation was not deemed necessary as the primary interest was in the linear association between continuous measurements.

(5) Mean absolute error (MAE): The MAE between Foot4D and Vicon measurements was calculated as an absolute index of measurement precision.

Although alternative regression-based approaches such as Passing–Bablok and Deming regression are available, we selected the Bland–Altman framework combined with ICC because (1) Bland–Altman plots provide direct visualization of systematic bias and proportional errors; (2) ICC quantifies reliability on an interpretable scale; and (3) this combination is the most widely adopted in markerless motion capture validation literature, facilitating cross-study comparability.

All statistical analyses were performed using participant-level means (n = 60), where each participant contributed a single mean value computed across 25 gait cycles (5 trials × 5 cycles). This analytical unit ensures statistical independence between observations and avoids artificial inflation of the degrees of freedom associated with treating nested strides as independent data points. The significance level was set at α = 0.05. All evaluated parameters are fully reported in Table 1 and Table 2; the text highlights key findings representative of the overall agreement patterns.

## 3. Results

### 3.1. Agreement Analysis of Spatiotemporal Parameters

**Figure 7:**
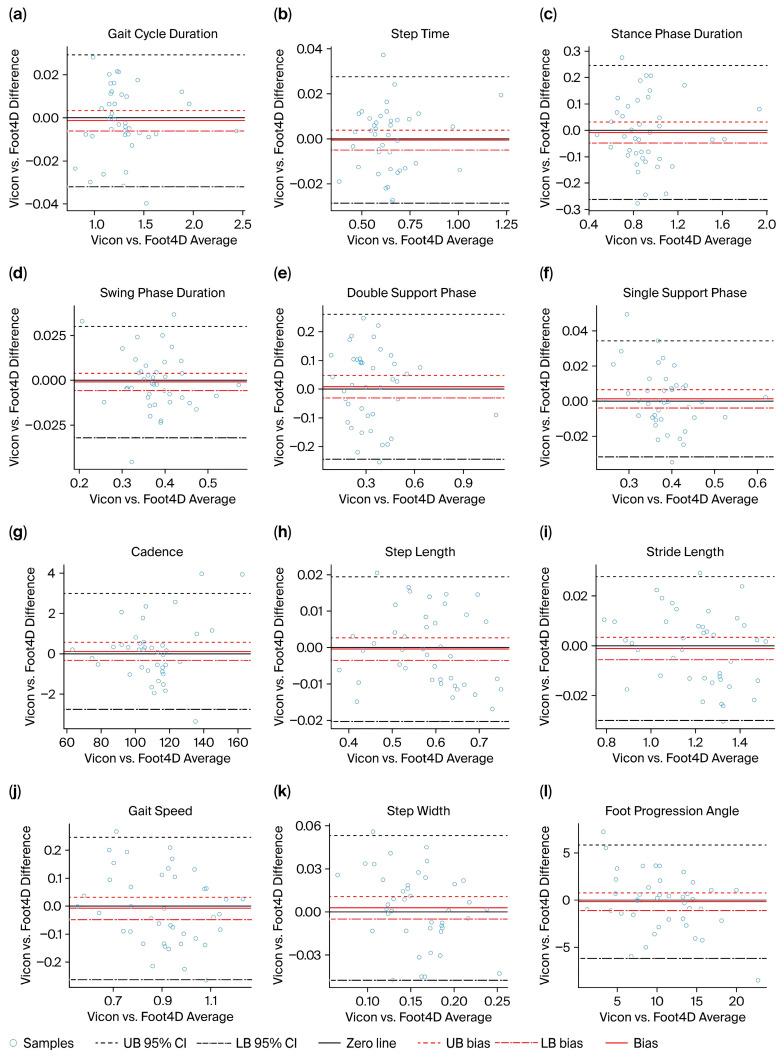
Bland–Altman plots illustrating the agreement of spatiotemporal gait parameters between the Foot4D and Vicon systems in patients with ankle injuries. The plots sequentially display the analysis results for 12 parameters: (**a**) gait cycle duration, (**b**) step time, (**c**) stance phase duration, (**d**) swing phase duration, (**e**) double-support phase, (**f**) single-support phase, (**g**) cadence, (**h**) step length, (**i**) stride length, (**j**) gait speed, (**k**) step width, and (**l**) foot progression angle. In each panel, the x-axis denotes the mean of the measurements from both systems, and the y-axis denotes the difference (Foot4D minus Vicon). The solid red line represents the mean difference (bias), and the dashed red lines indicate the 95% limits of agreement (LoA). All data points were closely clustered around the line of zero difference and lay within the 95% LoA, suggesting high agreement between the two systems across all evaluated parameters.

The Foot4D system demonstrated good-to-excellent agreement with the Vicon system across all 12 spatiotemporal gait parameters (ICC = 0.738–0.999, MAE = 0.009–0.108 m/s) (Table 1). The highest agreement was observed for gait cycle duration (ICC = 0.999, MAE = 0.012 s), step time (ICC = 0.996, MAE = 0.012 s), and stride length (ICC = 0.997, MAE = 0.012 m). Cadence (ICC = 0.997), step length (ICC = 0.994), and single-limb support time (ICC = 0.969) also exhibited excellent agreement. Walking speed (ICC = 0.738, MAE = 0.108 m/s) and foot progression angle (ICC = 0.817, MAE = 2.291°) showed relatively lower agreement compared with other parameters, yet remained within acceptable ranges. Bland–Altman analysis revealed that all data points fell within the 95% limits of agreement, with no evident proportional bias or heteroscedasticity observed (Figure 7).

### 3.2. Joint Kinematic Agreement Analysis

**Figure 8:**
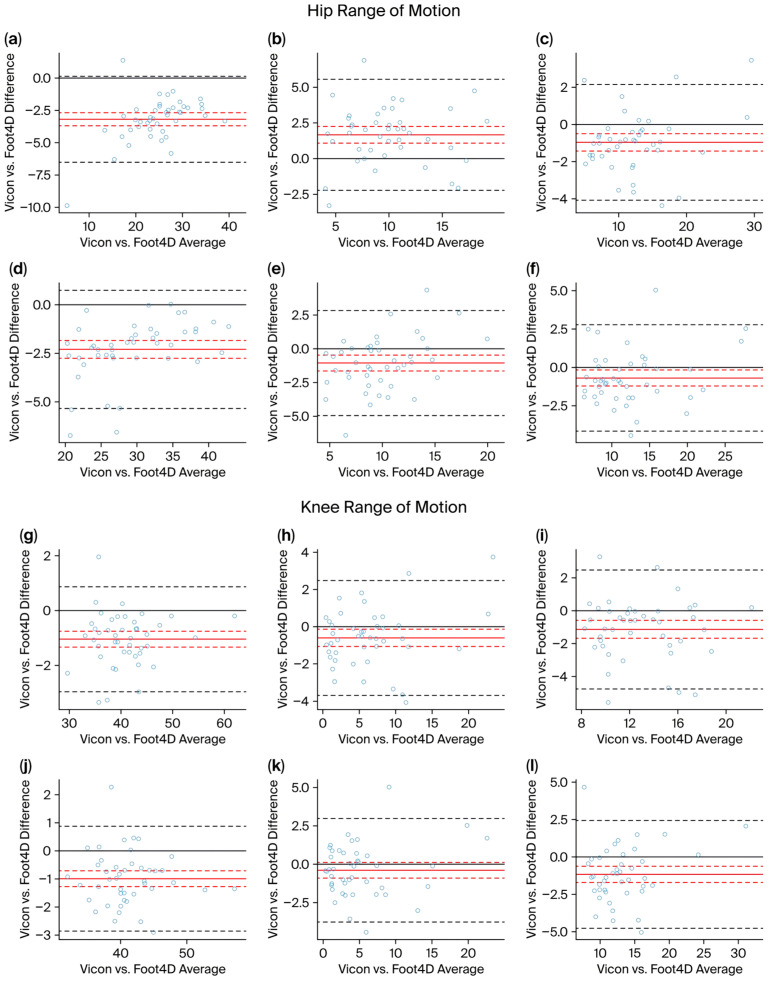

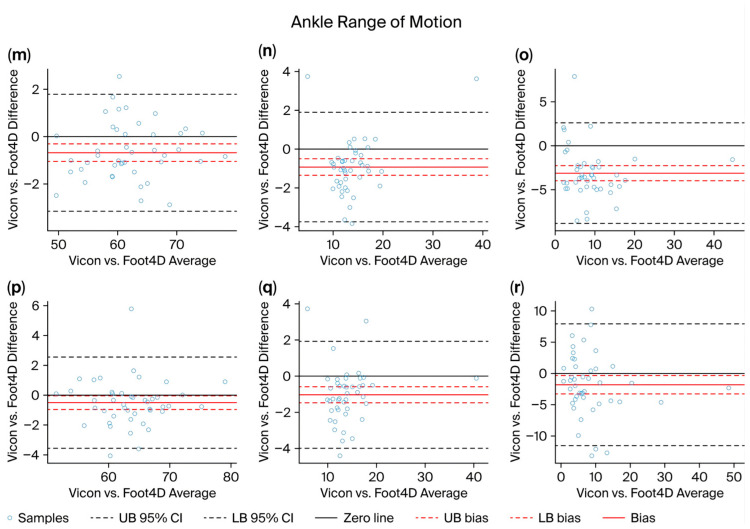
Bland–Altman plots illustrating the agreement of lower limb joint range of motion measurements between the Foot4D and Vicon systems in patients with ankle injuries. The affected side (upper panels) and healthy Side (lower panels) are presented separately for each joint and anatomical plane. In each panel, the x-axis denotes the mean of the measurements from both systems, and the y-axis denotes the difference (Foot4D minus Vicon). The solid red line represents the mean difference (bias), and the dashed red lines indicate the 95% limits of agreement (LoA). (**a**) Hip sagittal plane, affected side; (**b**) Hip coronal plane, affected side; (**c**) Hip transverse plane, affected side; (**d**) Hip sagittal plane, healthy side; (**e**) Hip coronal plane, healthy side; (**f**) Hip transverse plane, healthy side; (**g**) Knee sagittal plane, affected side; (**h**) Knee coronal plane, affected side; (**i**) Knee transverse plane, affected side; (**j**) Knee sagittal plane, healthy side; (**k**) Knee coronal plane, healthy side; (**l**) Knee transverse plane, healthy side; (**m**) Ankle sagittal plane, affected side; (**n**) Ankle coronal plane, affected side; (**o**) Ankle transverse plane, affected side; (**p**) Ankle sagittal plane, healthy side; (**q**) Ankle coronal plane, healthy side; (**r**) Ankle transverse plane, healthy side.

To evaluate the agreement between the Foot4D markerless system and the Vicon system in measuring three-dimensional lower limb joint kinematic parameters, this study calculated the intraclass correlation coefficients (ICC) and 95% limits of agreement (95% LoA) from Bland–Altman analysis for the primary angles of the hip, knee, and ankle joints in the sagittal, coronal, and transverse planes. Based on the gait cycle data from 60 subjects, the results are summarized in Table 2, with the corresponding Bland–Altman scatter distributions presented in Figure 8.

The agreement of three-dimensional lower limb joint kinematics exhibited a proximal-to-distal decreasing trend (Table 2). The hip joint demonstrated excellent agreement across all three anatomical planes (ICC = 0.934–0.970, MAE = 1.159–1.519°), with the highest consistency observed in the sagittal plane (ICC = 0.959–0.970). The knee joint showed good-to-excellent agreement in the sagittal and coronal planes (ICC = 0.941–0.976, MAE = 1.167–1.268°), whereas agreement in the transverse plane decreased to the lower threshold of good (ICC = 0.815–0.885, MAE = 1.549–1.753°).

The ankle joint sagittal plane (plantarflexion–dorsiflexion) demonstrated good agreement (affected side ICC = 0.857; healthy side ICC = 0.911; MAE = 2.299–3.250°), while the coronal plane (ICC = 0.801–0.813, MAE = 1.714–2.167°) and transverse plane (ICC = 0.816–0.835, MAE = 3.763–4.055°) decreased to good levels. Bland–Altman analysis revealed that the 95% limits of agreement for the ankle transverse plane were relatively wide, whereas the remaining parameters showed narrow limits centered around zero (Figure 8).

**Figure 9:**
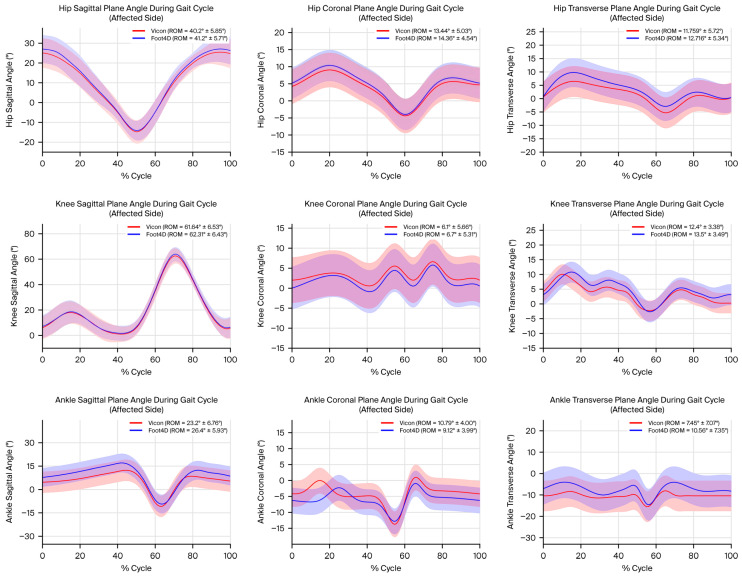
Comparison of three-dimensional lower limb joint kinematics between the Foot4D and Vicon systems during the gait cycle in patients with ankle injuries (affected side). This figure presents the ensemble average angle–time curves (0–100% normalized gait cycle) for the affected hip joint (first row), knee joint (second row), and ankle joint (third row) in the sagittal plane (left column), coronal plane (middle column), and transverse plane (right column). The red solid line represents measurements from the Vicon system, and the blue solid line represents measurements from the Foot4D system; shaded areas indicate ±1 standard deviation (SD) for each system. The legend indicates the range of motion (ROM) and its standard deviation for each joint in each plane. The angular trajectories of the unaffected lower limb joints are provided in Supplementary Figure S1.

To further visually assess the temporal measurement agreement between the Foot4D and Vicon systems across the gait cycle, Figure 9 presents the ensemble average angular trajectories of the affected lower limb hip, knee, and ankle joints in the sagittal, coronal, and transverse planes (0–100%, normalized gait cycle). The angular trajectories of the unaffected lower limb joints are provided in Supplementary Figure S1.

Integrating the temporal trajectory analysis from Figure 9 with the ROM agreement assessment results from Table 2, the Foot4D system demonstrated highly consistent dynamic measurement performance with the Vicon system across all three planes of the hip joint. The knee joint showed good concordance in angular trajectories in the sagittal and coronal planes. The overall waveform consistency in the ankle sagittal plane was acceptable; however, discernible limitations remained in dynamic tracking accuracy in the coronal and transverse planes.

## 4. Discussion

This study utilized the Vicon three-dimensional optical motion capture system as the reference standard and synchronously collected gait data from 60 patients with ankle injuries to systematically evaluate the accuracy of the Foot4D markerless non-contact gait analysis system in measuring lower limb three-dimensional kinematics and spatiotemporal gait parameters. The results revealed core findings at two levels. First, the Foot4D system demonstrated good-to-excellent agreement across all 12 spatiotemporal gait parameters (ICC range: 0.738–0.999), with temporal parameters (such as gait cycle duration and step time) showing particularly prominent consistency; the deviations from the Vicon system were clinically negligible. Second, in lower limb joint three-dimensional kinematic measurements, the Foot4D system exhibited a dual precision gradient characterized by a “proximal-to-distal decrement” and a “sagittal-to-non-sagittal decrement”. Specifically, the hip joint demonstrated optimal consistency (mean ICC ≈ 0.947), followed by the knee joint (mean ICC ≈ 0.921), with the ankle joint showing relatively lower consistency (mean ICC ≈ 0.839). Sagittal plane accuracy was generally superior to that of the coronal and transverse planes.

The high consistency demonstrated by the Foot4D system in spatiotemporal parameter measurements can be primarily attributed to the fact that the calculation of these parameters essentially relies on macroscopic geometric relationships of gait event detection and landmark spatial localization, rather than the fine capture of instantaneous joint angles. Temporal parameters such as gait cycle duration and step time are calibrated through extreme points (heel strike and toe-off) in the sagittal plane, and the Foot4D system, based on the SKEL model’s anatomical regression technique, can stably track the periodic displacement patterns of heel and toe landmarks, thereby achieving phase identification that is highly synchronized with Vicon marker trajectories. In this study, the ICC for gait cycle duration reached 0.999 with an MAE of merely 0.012 s, a precision level comparable to the automated gait analysis results based on MediaPipe Pose by Hii et al. [8] (stance time ICC = 0.945) and the study by Matsuda et al. [9] using the VisionPose 2D/3D system (step time ICC > 0.981), confirming the reliability of Foot4D in measuring temporal parameters.

Notably, the consistency for gait speed (ICC = 0.738) and foot progression angle (ICC = 0.817) was relatively lower than that for the other spatiotemporal parameters. Gait speed, as the ratio of stride length to step time, has an additive error source—when treadmill walking speed fluctuates due to patients’ subjective comfort adjustments, minute differences in stride length estimation between Foot4D and Vicon can be amplified. The foot progression angle, as a rotational parameter, is highly sensitive to the relative localization accuracy of foot landmarks in the transverse plane, and the anatomical regression precision of the SKEL model in the distal foot region (particularly the first and fifth metatarsophalangeal joints) is limited by soft tissue thickness and the probability of occlusion in this area, leading to increased variability in angle estimation. This finding is consistent with the trend reported by Roggio et al. [27], namely that rotational parameters (such as the foot progression angle) typically exhibit greater measurement noise in markerless systems than translational parameters.

Measurements obtained by the Foot4D system across all three anatomical planes of the hip joint achieved good-to-excellent consistency (ICC range: 0.934–0.970), with the mean MAE controlled within 1.2°. This excellent performance can be explained from the following technical perspectives: the hip joint, as the largest proximal joint of the lower limb, exhibits large motion amplitude with well-defined anatomical landmarks (regions such as the greater trochanter and anterior superior iliac spine are covered by relatively thin soft tissue), and the SKEL model’s anatomical regression matrix, trained on large-scale medical imaging data demonstrates high landmark localization precision in this region. Furthermore, sagittal plane flexion–extension motion of the hip joint is primarily defined by the relative angular change between the thigh and trunk, a geometric relationship that can be stably reconstructed using multi-view depth camera array with minimal influence from self-occlusion.

The measurement consistency of the knee joint was also at a high level, with ICC values for sagittal plane range of motion of 0.976 (affected side) and 0.956 (healthy side), and an MAE of approximately 1.2°. Sagittal plane motion of the knee joint represents the primary kinematic characteristic during the gait cycle, and the peak phase and amplitude of the flexion–extension angle curves demonstrated highly concordant temporal trajectories with those measured by Vicon in Figure 9. However, the consistency of knee joint transverse plane range of motion (ICC, 0.815–0.885) decreased compared with that of the sagittal plane. This phenomenon is similar to the findings of Matsuda et al. [9] when using the VisionPose multi-camera system (NEXT-SYSTEM Co., Ltd., Fukuoka, Japan) for tandem gait analysis—when the knee joint exhibits complex three-dimensional motion accompanied by tibial rotation, 2D image analysis systems struggle to accurately track rotational components in the transverse plane. Although Foot4D employs a multi-view depth camera array and outputs three-dimensional coordinates, in cases where the knee joint transverse plane motion amplitude itself is small (ROM approximately 12–14°), the relative contribution of system noise and minute landmark displacement to rotational angle calculation increases, leading to decreased consistency.

As the focus of this study, the ankle joint demonstrated a complex pattern in kinematic measurement consistency that warrants in-depth examination. The Foot4D system showed good consistency in ankle sagittal plane (plantarflexion–dorsiflexion) range of motion (affected side ICC = 0.857, healthy side ICC = 0.911), a result consistent with the central role of the ankle joint in gait function. Sagittal plane motion constitutes the majority of ankle ROM (approximately 23–28° in this study), and angle changes in this plane are primarily defined by the clear relative motion between the shank and foot, offering high geometric identifiability. However, the asymmetry of sagittal plane bias warrants attention: the Foot4D system systematically overestimated ankle sagittal plane range of motion (affected side bias = −3.188°, healthy side bias = −2.298°), meaning that the extreme angles of dorsiflexion and plantarflexion measured by Foot4D were both greater than those of Vicon. This systematic offset may be related to the skeletal regression characteristics of the SKEL model in the foot–ankle region. The SKEL model, based on standard SMPL anatomical regression, estimates the spatial orientation of the talus and calcaneus in the foot–ankle region through optimization fitting of shape parameters β and pose parameters θ, a regression process that may tend to generate greater foot–shank angle variation ranges than the marker-based model.

The consistency of ankle coronal plane (inversion–eversion) range of motion decreased to good levels (ICC ≈ 0.807), and the direction of bias showed inconsistency between the affected and healthy sides (affected side bias = +1.667°, healthy side bias = −1.054°). The challenge of coronal plane measurement stems from the complex anatomical geometry of the foot–ankle region: coronal plane motion of the ankle joint is not merely subtalar joint inversion/eversion but involves multi-articular coupled motion of the tibiotalar, subtalar, and transverse tarsal joints. The motion amplitude itself is small (approximately 9–11° in this study), such that minute landmark localization errors can produce significant angular deviations. Furthermore, ankle coronal plane angles are highly sensitive to the relative positions of landmarks along the coronal axis (*X*-axis), and the line-of-sight coverage of the multi-view depth cameras on the lateral aspect of the foot–ankle (particularly the lateral malleolus region) may suffer from local occlusion due to the contralateral limb or stance phase contact, leading to decreased point cloud fusion precision.

The consistency of transverse plane (internal–external rotation) range of motion was likewise good (ICC ≈ 0.826), with the largest MAE (affected side, 3.763°, healthy side, 4.055°). Transverse plane rotational motion represents the most challenging dimension to capture in ankle gait kinematics; its angle calculation depends on the relative directional change in shank and foot vector projections in the transverse plane. During the early stance phase (approximately 0–10% of the gait cycle), the rapid pronation of the foot at heel strike manifests as brief and minute angular fluctuations, the capture of which places high demands on both sampling frequency and spatial resolution. The 30 fps sampling frequency of Foot4D, after linear interpolation resampling to 100 Hz, may result in decreased temporal fidelity of high-frequency motion details. Wade et al. [14] noted in their review that current markerless motion capture systems generally exhibit lower accuracy in non-sagittal foot–ankle motion measurement than in the sagittal plane, a systematic technical bottleneck that aligns closely with the “lowest transverse plane precision” phenomenon observed in this study.

Taken together, the mean ICC of the Foot4D system across all three planes of the ankle joint was 0.839, falling within the “good” agreement range. According to the ICC grading criteria proposed by Koo and Li [31] (>0.9 excellent; 0.75–0.9 good; 0.5–0.75 moderate), ankle sagittal plane measurement meets the good standard, whereas the coronal and transverse planes fall at the lower threshold of the good range. For clinical gait assessment in patients with ankle injuries, sagittal plane kinematics (such as dorsiflexion limitation assessment) represents the most widely applied indicator, and the precision of Foot4D in this dimension can support clinical decision-making. However, for advanced application scenarios involving coronal and transverse plane motion pattern analysis (such as rotational stability assessment in ankle instability), system measurement errors must be interpreted with caution.

Placing the results of this study within the broader literature context of markerless motion capture technology facilitates positioning the relative advantages and technical boundaries of the Foot4D system. In studies based on 2D/3D pose estimation frameworks, Matsuda et al. [9] used the VisionPose AI engine to evaluate hip and knee joint angles in healthy adults during overground walking, reporting good consistency with ICC > 0.826 and a coefficient of determination R^2^ > 0.452. Foot4D approached this level, with hip sagittal ROM ICC = 0.934–0.970 and knee sagittal ROM ICC = 0.815–0.976 in patients with ankle injuies on a treadmill, although VisionPose remains essentially a 2D image analysis method with inherent limitations in transverse plane rotation tracking. Roggio et al. [27] combined MediaPipe BlazePose with a single smartphone camera to validate knee flexion–extension motion, reporting mean absolute errors of 4.10° (flexion) and 2.30° (extension), with a correlation coefficient ρ of 0.916, corresponding to a roughly two- to fourfold larger error than Foot4D’s knee sagittal ROM MAE of 1.16–1.17° in the present study. As in our findings, Roggio et al. observed significant ankle measurement errors, confirming the systematic difficulty of markerless systems in this anatomical region. Hii et al. [8] employed MediaPipe Pose for automated temporal parameter extraction, achieving a stance phase ICC = 0.945 in healthy gait, comparable to Foot4D’s stance time ICC=0.895 in a pathological population, with Foot4D’s gait cycle time ICC=0.999 indicating near-perfect temporal reconstruction. However, this system did not involve three-dimensional validation of joint angle kinematics.

An additional relevant comparison involves the native Azure Kinect Body Tracking SDK, which operates on the identical Azure Kinect DK depth cameras employed in the Foot4D system, thereby isolating the contribution of algorithmic architecture from hardware differences. Ma et al. [32] reported an RMSE of 11.9 ± 3.4° for knee sagittal angles (CMC = 0.87 ± 0.06) and 15.1 ± 6.5° for hip sagittal angles (CMC = 0.60 ± 0.34) using a dual Azure Kinect setup during overground walking in healthy adults (n = 5). Yeung et al. [33] reported an RMSE of 12.68 ± 5.12°for knee flexion/extension and 5.54 ± 2.43° for hip flexion/extension using a single Azure Kinect during treadmill walking (n = 20). Because RMSE disproportionately weights large discrepancies (mathematically, RMSE ≥ MAE for any error distribution; RMSE ≈ 1.253 × MAE under zero-mean Gaussian assumptions [34]), direct numerical comparison between RMSE-based and MAE-based studies requires caution. Nonetheless, the five- to tenfold reduction in knee kinematic error (Foot4D MAE 1.16–1.17° vs. Azure Kinect SDK RMSE 11.9–12.7°) and the comparable reduction in hip sagittal error (Foot4D MAE 1.16–1.52° vs. Azure Kinect SDK RMSE 5.5–15.1°) suggest that the integrated ViT-H/16 encoder, SKEL parametric model, and multi-view depth fusion pipeline of Foot4D confers measurable accuracy advantages over the native manufacturer SDK on identical hardware. These comparisons are indirect, however, involving different populations and walking conditions; direct head-to-head benchmarking under identical pathological conditions remains an important direction for future work.

Compared with the aforementioned systems relying on 2D pose estimation or consumer-grade depth sensors (such as Kinect), the Foot4D technical architecture offers two potential advantages. First, it employs Vision Transformer (ViT-H/16) as the image encoder, whose self-attention mechanism outperforms convolutional neural networks (CNN) in modeling long-range spatial dependencies, facilitating the extraction of more robust visual representations in regions rich in detail but with complex local features, such as the foot–ankle. Second, the SKEL parametric human model, through an anatomical regression matrix trained on medical imaging data, achieves penetrating mapping from body surface morphology to skeletal landmarks, which theoretically should improve the mitigation of soft tissue artifacts in the foot–ankle region during joint center localization. However, the results of this study indicate that although Foot4D performs excellently in spatiotemporal parameters and proximal joint measurements, the magnitude of precision improvement in non-sagittal ankle motion has not yet fully overcome the common technical bottleneck faced by markerless systems in this anatomical region. This finding suggests that fine kinematic measurement of the foot–ankle may require local imaging with higher spatial resolution, specialized regression models targeting foot–ankle anatomy, or the fusion of complementary sensing modalities such as inertial measurement units (IMUs) to compensate for the limitations of purely vision-based approaches.

This study, through synchronous comparison with the Vicon system, confirmed that Foot4D achieves good-to-excellent agreement for all spatiotemporal and hip–knee kinematic parameters in patients with ankle injuries. Although sagittal plane ankle kinematics demonstrated consistency adequate for clinical assessment, coronal- and horizontal plane measurements exhibited only moderate agreement, reflecting a common technical limitation of markerless systems in capturing subtle foot–ankle rotations. Several limitations should be acknowledged: the exclusion of patients with joint fibrosis, severe mobility limitation, or obesity; the potential smoothing of high-frequency motion details attributable to linear interpolation resampling from 60 Hz to 100 Hz; the joint angles reported in this study were computed using a plane-specific vector projection method rather than full ISB-recommended segment-fixed local coordinate systems with Cardan/Euler decomposition, which may not fully account for segmental torsion or out-of-plane rotation cross-talk; the single-center cross-sectional design, which precludes assessment of test–retest and multicenter reliability; and the intra-session repeatability of each individual system was not formally assessed in this study, although the repeated trial design (five trials per participant) would support such analysis in future work. Additionally, direct quantitative benchmarking of Foot4D against other established markerless systems (e.g., Azure Kinect Body Tracking SDK, OpenPose, and MediaPipe) under identical pathological conditions was not performed in this study; such head-to-head inter-markerless comparison will be addressed in our ongoing follow-up work. Future efforts should also focus on implementing full ISB-compliant LCS-based Cardan analysis for rigorous multiplanar joint kinematic assessment, in addition to optimizing the SKEL foot–ankle regression matrix, integrating wearable IMUs to enhance non-sagittal rotational accuracy, conducting multicenter prospective rehabilitation studies, and developing lightweight home-deployment solutions for remote gait monitoring.

## 5. Conclusions

In conclusion, through synchronous comparison with the Vicon three-dimensional motion capture system, this study systematically validated the measurement accuracy of the Foot4D markerless non-contact gait analysis system in patients with ankle injuries. The Foot4D system demonstrated good-to-excellent agreement across all spatiotemporal gait parameters and three-dimensional kinematic parameters of the hip and knee joints, confirming its technical reliability as a clinical gait assessment tool. In ankle kinematic measurements, sagittal plane (plantarflexion–dorsiflexion) agreement was good, meeting basic clinical assessment requirements. However, coronal plane (inversion–eversion) and horizontal plane (internal–external rotation) agreement were at the lower bound of the good range, suggesting that markerless vision-based systems still face common technical challenges in fine three-dimensional motion capture of the foot–ankle region. Leveraging its markerless, non-contact, and operationally convenient technical characteristics, Foot4D provides a novel tool with clinical translational prospects for gait function assessment in patients with ankle injuries, particularly demonstrating clear application value in monitoring spatiotemporal parameters and proximal joint kinematics. Future research should focus on targeted optimization of algorithms for the foot–ankle region and multi-scenario clinical validation to promote the deep integration of markerless motion capture technology from laboratory settings into clinical practice.

## Figures and Tables

**Table 1:** Comparison of spatiotemporal parameter measurements between the Foot4D and Vicon systems (mean ± standard deviation) and agreement analysis.

Characteristic	Vicon	Foot4D	*p*-Value	ICC	MAE	Bias	95% CI of Bias	95% LoA
Gait cycle (s)	1.292 ± 0.290	1.293 ± 0.290	0.589	0.999	0.012	−0.001	[−0.006, 0.003]	[−0.032, 0.029]
Step time (s)	0.645 ± 0.151	0.645 ± 0.150	0.807	0.996	0.012	−0.001	[−0.005, 0.004]	[−0.029, 0.027]
Stance time (s)	0.911 ± 0.279	0.920 ± 0.281	0.675	0.895	0.105	−0.009	[−0.048, 0.031]	[−0.262, 0.245]
Swing time (s)	0.379 ± 0.064	0.380 ± 0.066	0.713	0.971	0.012	−0.001	[−0.006, 0.004]	[−0.032, 0.030]
Double support (s)	0.341 ± 0.177	0.333 ± 0.193	0.678	0.762	0.108	0.008	[−0.031, 0.048]	[−0.244, 0.261]
Single support (s)	0.383 ± 0.065	0.382 ± 0.070	0.610	0.969	0.013	0.001	[−0.004, 0.007]	[−0.032, 0.034]
Cadence (steps/min)	110.121 ± 18.468	110.007 ± 18.164	0.620	0.997	1.047	0.114	[−0.333, 0.561]	[−2.751, 2.979]
Step length (m)	0.580 ± 0.094	0.581 ± 0.096	0.795	0.994	0.009	−0.000	[−0.004, 0.003]	[−0.020, 0.019]
Stride length (m)	1.205 ± 0.184	1.206 ± 0.188	0.648	0.997	0.012	−0.001	[−0.006, 0.003]	[−0.030, 0.028]
Walking speed (m/s)	0.901 ± 0.160	0.910 ± 0.194	0.694	0.738	0.108	−0.008	[−0.048, 0.032]	[−0.263, 0.247]
Step width (m)	0.157 ± 0.046	0.159 ± 0.037	0.480	0.812	0.021	0.003	[−0.005, 0.011]	[−0.048, 0.053]
Foot angle (°)	10.715 ± 5.565	10.542 ± 4.445	0.721	0.817	2.291	−0.173	[−1.115, 0.769]	[−6.205, 5.859]

**Table 2:** Comparison of lower limb joint range of motion parameters between the Foot4D and Vicon systems (mean ± standard deviation) and agreement analysis.

Characteristic	Limb	Vicon	Foot4D	*p*	ICC	MAE	Bias	95% CI of Bias	95% LoA
	Sagittal plane Affected side	40.188 ± 5.791	41.232 ± 5.711	<0.001	0.970	1.159	−1.045	[−1.332, −0.757 ]	[−2.953, 0.864]
Hip	Coronal plane Affected side	13.436 ± 5.028	14.359 ± 4.540	<0.001	0.938	1.352	−0.922	[−1.348, −0.497 ]	[−3.745, 1.900]
	Transverse plane Affected side	11.759 ± 5.716	12.716 ± 5.339	<0.001	0.945	1.476	−0.957	[−1.427, −0.488]	[−4.070, 2.155]
	Sagittal plane Healthy side	40.583 ± 4.692	41.573 ± 4.764	<0.001	0.959	1.163	−0.989	[−1.270, −0.708 ]	[−2.854, 0.876]
	Coronal plane Healthy side	13.593 ± 5.014	14.624 ± 4.904	<0.001	0.934	1.418	−1.031	[−1.477, −0.585]	[−3.991, 1.929]
	Transverse plane Healthy side	11.980 ± 5.396	12.669 ± 5.064	0.013	0.936	1.519	−0.689	[−1.213, −0.166]	[−4.160, 2.781]
Knee	Sagittal plane Affected side	61.635 ± 6.529	62.314 ± 6.432	<0.001	0.976	1.167	−0.679	[−1.052, −0.306]	[−3.153, 1.794]
	Coronal plane Affected side	6.073 ± 5.663	6.675 ± 5.313	0.015	0.954	1.268	−0.602	[−1.067, −0.138 ]	[−3.685, 2.481]
	Transverse plane Affected side	12.368 ± 3.380	13.501 ± 3.488	<0.001	0.815	1.549	−1.133	[−1.677, −0.589]	[−4.741, 2.475]
	Sagittal plane Healthy side	63.266 ± 5.448	63.771 ± 5.422	0.037	0.956	1.163	−0.505	[−0.966, −0.044]	[−3.562, 2.552]
	Coronal plane Healthy side	4.869 ± 5.236	5.264 ± 4.957	0.135	0.941	1.363	−0.395	[−0.904, 0.113]	[−3.769, 2.978]
	Transverse plane Healthy side	12.452 ± 4.591	13.616 ± 4.239	<0.001	0.885	1.753	−1.164	[−1.707, −0.621]	[−4.765, 2.437]
Ankle	Sagittal plane Affected side	23.197 ± 6.756	26.385 ± 5.926	<0.001	0.857	3.250	−3.188	[−3.689, −2.687]	[−6.512, 0.136]
	Coronal plane Affected side	10.785 ± 4.000	9.118 ± 3.993	<0.001	0.801	2.167	1.667	[1.080, 2.253]	[−2.224, 5.558]
	Transverse plane Affected side	7.448 ± 7.067	10.558 ± 7.351	<0.001	0.835	3.763	−3.110	[−3.972, −2.248]	[−8.827, 2.606]
	Sagittal plane Healthy side	28.274 ± 6.698	30.572 ± 6.000	<0.001	0.911	2.299	−2.298	[−2.757, −1.839]	[−5.343, 0.747]
	Coronal plane Healthy side	9.410 ± 3.947	10.464 ± 3.223	0.001	0.813	1.714	−1.054	[−1.649, −0.468]	[−4.943, 2.835]
	Transverse plane Healthy side	7.541 ± 8.230	9.340 ± 8.903	0.021	0.816	4.055	−1.798	[−3.266, −0.330]	[−11.535, 7.938]

Note: ICC (2,1), two-way random-effects model with absolute agreement; 95% CI: 95% confidence interval of the mean bias; mean bias (±1.96SD): mean bias and 95% limits of agreement (LoA) from Bland–Altman analysis.

## Data Availability

The data presented in this study are available on request from the corresponding authors (Qinwei Guo or Shuang Ren). The data are not publicly available due to privacy restrictions involving patient medical records and requirements stipulated by the institutional ethics committee.

## Supplementary Materials

The following supporting information can be downloaded at: https://www.mdpi.com/article/10.3390/s26144579/s1, Figure S1: Comparison of three-dimensional lower limb joint kinematics between the Foot4D and Vicon systems during the gait cycle in patients with ankle injuries (healthy side). This figure presents the ensemble average angle–time curves (0–100% normalized gait cycle) for the unaffected hip joint (first row), knee joint (second row), and ankle joint (third row) in the sagittal plane (left column), coronal plane (middle column), and transverse plane (right column). The red solid line represents measurements from the Vicon system, and the blue solid line represents measurements from the Foot4D system; shaded areas indicate ±1 standard deviation (SD) for each system. The legend in-dicates the range of motion (ROM) and its standard deviation for each joint in each plane.

## Abbreviations

The following abbreviations are used in this manuscript:

2D	Two-dimensional
3D	Three-dimensional
CI	Confidence interval
CNN	Convolutional neural network
fps	Frames per second
HS	Heel strike
ICC	Intraclass correlation coefficient
IMU	Inertial measurement unit
ISB	International Society of Biomechanics
LoA	Limits of agreement
MAE	Mean absolute error
ROM	Range of motion
SD	Standard deviation
SKEL	Skeletal Kinematics Enveloped by a Learned body model
SMPL	Skinned Multi-Person Linear model
SPSS	Statistical Package for the Social Sciences
TO	Toe-off
ViT	Vision transformer

## Appendix A. Mathematical Formulations of Camera Calibration

The Foot4D multimodal acquisition system developed in this study employs a two-stage, high-precision spatial alignment strategy: factory-level intrinsic calibration and scene-geometry-prior-based in situ extrinsic self-calibration. This approach ensures submillimeter accuracy in 3D reconstruction while enabling seamless one-click initiation of clinical data acquisition.

### Appendix A.1. Factory Intrinsic Calibration

During the system manufacturing phase, the internal optical parameters of all cameras were pre-calibrated using a high-precision checkerboard pattern combined with Zhang’s calibration method. For any i-th camera in the system, the intrinsic matrix $K_{i}$ is defined as:

$$K_{i}=\left(\begin{array}{ccc} f_{x,i} & 0 & c_{x,i}\\ 0 & f_{y,i} & c_{y,i}\\ 0 & 0 & 1 \end{array}\right)$$

where $f_{x,i}$ and $f_{y,i}$ denote the equivalent focal lengths of the camera, and $c_{x,i}$ and $c_{y,i}$ represent the principal point coordinates. Simultaneously, the system computed and compensated for both radial and tangential lens distortions to ensure pixel mapping accuracy across the peripheral field of view.

### Appendix A.2. In Situ Extrinsic Self-Calibration Based on the Treadmill Frame

To unify the six cameras distributed at different viewpoints into a single global coordinate system (world coordinate system, W), this system abandons the conventional dynamic wand calibration and instead adopts the treadmill’s rigid metal frame as a global spatial anchor. The three-dimensional CAD geometric model of the treadmill frame is pre-stored in the system as prior knowledge. When the operator clicks the “Start Recording” button, the extrinsic self-calibration algorithm is triggered instantaneously.

**Feature extraction and matching:** The RGB-D sensors of each camera synchronously capture the scene. Through edge detection operators and 3D point cloud segmentation algorithms, the geometric primitives (such as 3D line segments and corner points) of the treadmill frame in the field of view are automatically extracted.

**Rigid body transformation solution:** Let $P_{W}$ be the known coordinates of a three-dimensional feature point on the treadmill frame in the global coordinate system, and let $P_{C_{i}}$ be the observed coordinates of this point in the local coordinate system of the i-th camera. The following rigid body transformation relationship exists between them:

$$P_{C_{i}}=R_{i}P_{W}+T_{i}$$

where $R_{i}$ is a 3×3 rotation matrix, and $T_{i}$ is a 3×1 translation vector.

Nonlinear optimization: The system precisely solves for the extrinsic parameters by minimizing the three-dimensional spatial reprojection error of all matched feature points. The objective function is iteratively optimized using the Levenberg–Marquardt (LM) algorithm:

$$\arg\underset{R_{i},T_{i}}{\min}\sum_{j=1}^{N}{\left|p_{i,j}-\pi \left(K_{i}\left(R_{i}P_{W,j}+T_{i}\right)\right)\right|}^{2}$$

where N is the total number of valid treadmill frame feature points extracted, $p_{i,j}$ is the two-dimensional observed coordinate of the feature point on the image plane, and $\pi \left(\cdot \right)$ is the perspective projection function. For the direct 3D point clouds acquired by the depth cameras, the system simultaneously incorporates the Iterative Closest Point (ICP) algorithm for fine registration of multi-viewpoint point clouds.

## Appendix B. SKEL-Based Markerless Kinematic Reconstruction Algorithm

This study abandons traditional two-dimensional keypoint detection schemes and instead adopts a three-dimensional parametric human body model based on SKEL (Skeletal Kinematics Enveloped by a Learned body model), combined with multi-view depth point clouds, to achieve high-precision mapping from surface morphology to the internal anatomical skeletons. The entire markerless algorithmic framework consists of three mathematical modules: parametric modeling, nonlinear data fitting, and anatomical skeleton regression.

### Appendix B.1. Parametric Human Body Morphology and Kinematic Modeling

The SKEL model is built upon the statistical body model SMPL (Skinned Multi-Person Linear model), which is driven by shape parameters and pose parameters to generate the three-dimensional human body mesh. Let $\beta \in R^{\left|\beta \right|}$ denote the shape parameters controlling body stature and physique (typically the principal component weights after PCA dimensionality reduction), and let $\theta \in R^{\left|\theta \right|}$ denote the pose parameters controlling joint rotations.

The three-dimensional surface mesh model $M\left(\beta ,\theta \right)$ generated by the system can be expressed through the linear blend skinning (LBS) function $W\left(\cdot \right)$:

$$M\left(\beta ,\theta \right)=W\left(T\left(\beta ,\theta \right),J\left(\beta \right),\theta ,W\right)$$

where $T\left(\beta ,\theta \right)$ is the template mesh incorporating blended weights for shape and pose deformations; $J\left(\beta \right)$ is the initial three-dimensional joint centers regressed from the shape parameters; and W is the skinning weight matrix used to compute the influence of skeletal motion on surface soft tissue deformation.

### Appendix B.2. Nonlinear Optimization Based on Multi-View Point Clouds

After acquiring the globally fused three-dimensional observed point cloud $P=p_{1},p_{2},\ldots ,p_{N}$ from the six cameras, the system inversely solves for the optimal shape and pose parameters of the subject in the current frame by minimizing the energy objective function $E\left(\beta ,\theta \right)$. The objective function is defined as a weighted sum of the data term and the prior term:

$$E\left(\beta ,\theta \right)=\lambda_{data}E_{data}+\lambda_{prior}E_{prior}$$

**Data fitting term (**$E_{data}$**):** The Chamfer distance or point-to-surface distance is employed to constrain the generated SKEL model surface $M\left(\beta ,\theta \right)$ to closely fit the actual point cloud P captured by the multi-camera system:

$$E_{data}\left(\beta ,\theta \right)=\sum_{p_{i}\in P}{\underset{v_{j}\in M\left(\beta ,\theta \right)}{\min}\left|p_{i}-v_{j}\right|}_{2}^{2}$$

**Biomechanical prior term (**$E_{prior}$**)**: To prevent abnormal poses caused by missing depth camera point clouds (e.g., due to occlusion), the system introduces a latent pose prior trained on large-scale motion capture datasets, along with joint angle limits:

$$E_{prior}=\left|\beta \right|2^{2}+Lpose\left(\theta \right)+E_{collision}$$

where $Lpose\left(\theta \right)$ penalizes anomalous joint rotations that violate human anatomical limits, and $E_{collision}$ is used to prevent self-penetration of the model. This nonlinear least-squares problem is solved iteratively using the Levenberg–Marquardt (LM) algorithm.

### Appendix B.3. Precise Mapping from Surface to Anatomical Skeleton

The “joints” extracted by conventional computer vision models are often geometric centers of surface features rather than true biomechanical rotation centers. The core advantage of the SKEL model lies in its built-in anatomical regressor, optimized using high-precision medical imaging datasets such as BioAMASS.

Let $J_{anat}$ be the final output three-dimensional anatomical landmark coordinates conforming to the International Society of Biomechanics (ISB) standards. The calculation formula is:

$$J_{anat}=R_{anat}M\left(\beta^{*},\theta^{*}\right)$$

where $\beta^{*}$ and $\theta^{*}$ are the optimal parameters obtained from the above optimization, and $R_{anat}$ is the sparse regression matrix. This matrix is trained through the registration of extensive MRI/CT data with surface scan data and is capable of penetrating soft tissues (such as the plantar fat pads and calf muscles) to precisely derive the three-dimensional spatial coordinates of deep skeletal landmarks, including the medial and lateral femoral epicondyles and the medial and lateral malleoli.

## Appendix C. Three-Dimensional Joint Angle Calculation Model

For lower limb kinematic analysis, this study constructs a three-dimensional spatial vector angle model based on the high-precision three-dimensional anatomical landmarks output by the SKEL model. Unlike traditional two-dimensional video analysis, the Foot4D system performs spatial vector operations directly in the global three-dimensional coordinate system O−XYZ, combined with anatomical plane projections, to obtain true clinical joint range of motion (ROM).

### Appendix C.1. Three-Dimensional Spatial Base Angle Calculation

Taking the knee joint as an example, let the three-dimensional spatial coordinates of the hip joint center, knee joint center, and ankle joint center extracted by the system at time t be defined as follows: hip joint point: $P_{H}=\left(x_{h},y_{h},z_{h}\right)$; knee joint point: $P_{K}=\left(x_{k},y_{k},z_{k}\right)$; and ankle joint point: $P_{A}=\left(x_{a},y_{a},z_{a}\right)$.

Using the knee joint $P_{K}$ as the vertex, the thigh spatial vector $\vec{u}$ and shank spatial vector $\vec{v}$ are constructed:

$$\vec{u}=P_{H}-P_{K}=\left(x_{h}-x_{k},y_{h}-y_{k},z_{h}-z_{k}\right)$$

$$\vec{v}=P_{A}-P_{K}=\left(x_{a}-x_{k},y_{a}-y_{k},z_{a}-z_{k}\right)$$

According to the dot product principle of three-dimensional spatial vectors, the absolute angle $\theta_{3D}$ of the knee joint in three-dimensional space can be precisely solved by the following equation:

$$\cos\theta_{3D}=\frac{\vec{u}\cdot \vec{v}}{\left|\vec{u}\right|\left|\vec{v}\right|}=\frac{u_{x}v_{x}+u_{y}v_{y}+u_{z}v_{z}}{\sqrt{u_{x}^{2}+u_{y}^{2}+u_{z}^{2}}\sqrt{v_{x}^{2}+v_{y}^{2}+v_{z}^{2}}}$$

$$\theta_{3D}=\arccos\left(\frac{\vec{u}\cdot \vec{v}}{\left|\vec{u}\right|\left|\vec{v}\right|}\right)$$

### Appendix C.2. Clinical Angle Projection Based on Anatomical Planes

To conform to the clinical medical definitions of joint flexion–extension and varus–valgus, the system projects the aforementioned three-dimensional vectors onto specific anatomical planes. Assuming that the Y−Z plane of the system corresponds to the human sagittal plane, the projected vectors of the thigh and shank on the sagittal plane are $\vec{u}sag=\left(0,y_{h}-y_{k},z_{h}-z_{k}\right)$ and $\vec{v}sag=\left(0,y_{a}-y_{k},z_{a}-z_{k}\right)$, respectively. At this point, the most clinically relevant knee joint flexion angle (flexion angle, $\theta_{flex}$) is calculated as:

$$\theta_{flex}=180^{\circ }-\arccos\left(\frac{\vec{u}sag\cdot \vec{v}sag}{\left|\vec{u}sag\right|\left|\vec{v}sag\right|}\right)$$

As shown in Figure 6, in the global coordinate system composed of the coronal axis (X), sagittal axis (Y), and vertical axis (Z), the SKEL model precisely localizes the three-dimensional spatial coordinates of the hip joint $P_{H}$, knee joint $P_{K}$, and ankle joint $P_{A}$. Using the knee joint $P_{K}$ as the rotation center, the femur (thigh) and tibia (shank) are abstracted as spatial vectors $\vec{u}$ and $\vec{v}$ with definite directions and lengths. The orange dashed arc in the figure intuitively indicates the absolute true angle $\theta_{3D}$ between the two vectors in three-dimensional space. Compared with traditional two-dimensional video capture technology, this pure three-dimensional spatial vector-based calculation model eliminates projection distortion errors caused by camera perspective deviation or out-of-plane human motion, thereby providing a rigorous mathematical and geometric foundation for the subsequent precise decomposition of kinematic data into specific anatomical planes (such as sagittal plane flexion–extension and coronal plane varus–valgus).

### Appendix C.3. Distinction Between the 3D Vector Projection Method and ISB-Recommended Cardan Analysis

The ISB-recommended approach for multiplanar joint angles employs segment-fixed local coordinate systems (LCSs) with a Cardan rotation sequence (typically the Z-X-Y order). In this framework, the rotation matrix R describing the distal segment relative to the proximal segment is decomposed as:

$$R=R_{z}\left(\alpha \right)R_{x}\left(\beta \right)R_{y}\left(\gamma \right)$$

where α, β, and γ correspond to transverse-, coronal-, and sagittal plane rotations, respectively.

The 3D spatial vector projection method employed in Foot4D takes a different path. Let $v_{\mathrm{prox}\;}$ and $v_{\mathrm{dist}\;}$ be the proximal and distal segment vectors in the global coordinate system. The 3D absolute angle is:

$$\theta_{3D}=arccos\left(\left(v_{\mathrm{prox}}\cdot v_{\mathrm{dist}}\right)/\left(\left|v_{\mathrm{prox}}\right|\left|v_{\mathrm{dist}}\right|\right)\right).$$

Clinical planar angles are obtained by projecting the segment vectors onto anatomical planes before computing the angle. For the sagittal plane:

$$\left(Y-Z\;\mathrm{plane}\;\right):\theta_{\;\mathrm{sagittal}\;}\;plane\;arccos\left(\left(v_{\mathrm{prox}}^{\mathrm{sag}}\cdot v_{\mathrm{dist}}^{\mathrm{sag}}\right)/\left(\left|v_{\mathrm{prox}}^{\mathrm{sag}}\right|\left|v_{\mathrm{dist}}^{\mathrm{sag}}\right|)\right),\right.$$

where $v_{\mathrm{prox}}^{\mathrm{sag}}=\left[0,v_{y}^{\mathrm{prox}},v_{z}^{\mathrm{prox}}\right]$ and $v_{\mathrm{dist}}^{\mathrm{sag}}=\left[0,v_{y}^{\mathrm{dist}},v_{z}^{\mathrm{dist}}\right].$ Coronal- and transverse plane angles are computed analogously by zeroing the respective coordinate.

It is important to emphasize that these planar projection ROMs are not equivalent to ISB Cardan angles. While both methods quantify multiplanar joint motion, they differ in their underlying mathematical assumptions: Cardan sequences explicitly model segment-fixed coordinate transformations and are sensitive to rotation order and gimbal lock when the middle angle approaches 90°, whereas vector projections operate directly on SKEL-output ISB-compliant 3D landmark coordinates without intermediate LCS construction. The vector projection method was selected for the present study because (1) it provides clinically interpretable sagittal-, coronal-, and transverse plane ROM indices; (2) it avoids additional instability from estimating full segment-fixed local coordinate systems in markerless data; and (3) it ensures matched computational definitions between Foot4D and Vicon for concurrent agreement assessment.

Compared with ISB-recommended Cardan analysis, the vector projection method offers three practical characteristics for the present concurrent validity study: (1) it does not require construction of intermediate segment-fixed local coordinate systems, which can be unstable in markerless data; (2) it produces unambiguous planar angles without rotation order dependency; and (3) it directly utilizes SKEL’s ISB-compliant 3D landmark coordinates.

Two important limitations must be acknowledged: (1) segmental torsion (e.g., femoral anteversion) is handled implicitly through the SKEL anatomical regression matrix rather than explicitly via individualized segment-fixed LCS, which may introduce geometric cross-talk in subjects with atypical anatomy; and (2) out-of-plane rotation coupling is not explicitly modeled as in Cardan sequences, meaning that for large multiplanar excursions or abnormal gait patterns, the projection angles may not fully capture the true three-dimensional joint kinematics. For applications requiring rigorous multiplanar joint kinematic assessment (e.g., pre-surgical planning or detailed biomechanical modeling), full ISB-recommended LCS-based Cardan analysis remains the preferred approach and should be implemented in future iterations of the Foot4D system.

## Appendix D. Mathematical Models for Spatiotemporal Parameter Calculation

### Appendix D.1. Data Processing and Gait Event Detection

The spatiotemporal parameters in this study were computed offline based on three-dimensional kinematic data. Since the subjects walked on a treadmill, the vertical displacement (*Z*-axis) of the foot exhibited no significant extremum features due to the support of the treadmill belt, and conventional force plate data were unavailable. Therefore, this study employed an “extremum method” based on the anterior–posterior (*Y*-axis) trajectories of the foot markers to identify gait events.

Specifically, the heel strike (HS) event was defined as the instant when the heel marker reached the most anterior position during the gait cycle, i.e., the positive local maximum of the marker in the anterior–posterior direction (*Y*-axis) of the reference coordinate system. The toe-off (TO) event was defined as the instant when the toe marker reached the most posterior position moving with the treadmill belt, i.e., the negative local minimum of the marker in the *Y*-axis. Prior to extremum searching, all three-dimensional marker trajectories were smoothed using a fourth-order zero-phase Butterworth low-pass filter with a cutoff frequency of 6 Hz to eliminate high-frequency soft tissue artifacts and instrumental noise that could interfere with gait event detection.

### Appendix D.2. Quantitative Formulations of Spatiotemporal Parameters

Following successful extraction of consecutive gait events, the calculation of all spatiotemporal parameters was based on the relative spatial positions in the local coordinate system. Let tHS1 and tHS2 denote the timestamps of two consecutive ipsilateral (e.g., right) heel strikes, tcHS denotes the contralateral (left) heel strike occurring within this interval, and tTO denotes the ipsilateral toe-off time.

#### Appendix D.2.1. Temporal Parameters

Gait cycle time (Tcyc) was defined as the time difference between two consecutive ipsilateral heel strikes:

Tcyc = tHS2 − tHS1

Stance time (Tst) was defined as the time difference from ipsilateral heel strike to toe-off:

Tst = tTO − tHS1

Stance phase percentage was calculated as:

$$P_{st}=\frac{T_{st}}{T_{cyc}}\times 100$$

Double-support time (Tds) was defined as the total duration during which both feet simultaneously contacted the treadmill belt within a gait cycle, i.e., from one heel strike (tHS) to the contralateral toe-off (tTO):

$$T_{ds}=\left(t_{L_{T}O}-t_{R_{H}S}\right)+\left(t_{R_{T}O}-t_{L_{H}S}\right)$$

Single-support time (Tss) was defined as the duration during which only one lower limb independently supported the body weight, obtained by subtracting the double-support time involving that side from the total stance time:

$$T_{ss}=T_{st}-T_{ds}$$

Cadence (C), a fundamental temporal parameter, was converted from gait cycle time to steps per minute:

$$C=\frac{120}{T_{cyc}}$$

#### Appendix D.2.2. Spatial Parameters

The calculation of spatial parameters excluded absolute displacement errors caused by treadmill belt motion and employed the relative Euclidean distance between bilateral foot markers at the same instant for quantification. Let (xR, yR) and (xL, yL) denote the coordinates of the right and left heel markers in the horizontal plane (X-Y plane), respectively.

Right step length (lR) was defined as the straight-line distance between the bilateral heel markers at the instant of right heel strike (tHS1):

$$l_{R}=\sqrt{{\left(x_{R}\left(t_{HS1}\right)-x_{L}\left(t_{HS1}\right)\right)}^{2}+{\left(y_{R}\left(t_{HS1}\right)-y_{L}\left(t_{HS1}\right)\right)}^{2}}$$

Similarly, left step length (lL) was the horizontal distance between the bilateral heels at the instant of left heel strike (tcHS):

$$l_{L}=\sqrt{{\left(x_{L}\left(t_{cHS}\right)-x_{R}\left(t_{cHS}\right)\right)}^{2}+{\left(y_{L}\left(t_{cHS}\right)-y_{R}\left(t_{cHS}\right)\right)}^{2}}$$

Stride length (L) was calculated as the sum of consecutive left and right step lengths:

L = lR + lL

Step width (w) was defined as the absolute distance between the bilateral heel markers in the coronal plane (*X*-axis, i.e., mediolateral direction) at the instant of heel strike. This reflects the compensatory strategy adopted by patients to increase the support base:

$$w=\left|x_{R}\left(t_{HS1}\right)-x_{L}\left(t_{HS1}\right)\right|$$

Foot progression angle (θ) was defined as the angle between the line connecting the toe marker and the heel marker and the direction of progression (*Y*-axis) at the instant of heel strike:

$$\theta =\arctan\left(\frac{\left|x_{TOE}\left(t_{HS1}\right)-x_{HEE}\left(t_{HS1}\right)\right|}{\left|y_{TOE}\left(t_{HS1}\right)-y_{HEE}\left(t_{HS1}\right)\right|}\right)\times \frac{180}{\pi }$$

### Appendix D.2.3. Velocity Parameters

Finally, equivalent walking speed (v) was obtained from the ratio of stride length to gait cycle time:

$$v=\frac{L}{T_{cyc}}$$
