# Supplementary material for: Validation of a Markerless Non-Contact Gait Analysis System for Three-Dimensional Gait Kinematics in Patients with Ankle Injuries: A Concurrent Comparison Study with the Vicon Three-Dimensional Motion Capture System
Source: Sensors (Basel). 2026 Jul 19;26(14):4579. doi: 10.3390/s26144579 (PMC13418591; doi:10.3390/s26144579)
Supplement: Supplementary file 1 [file sensors-26-04579-s001.zip › sensors-4407172-Supplementary Materials.pdf]

Supplementary Materials

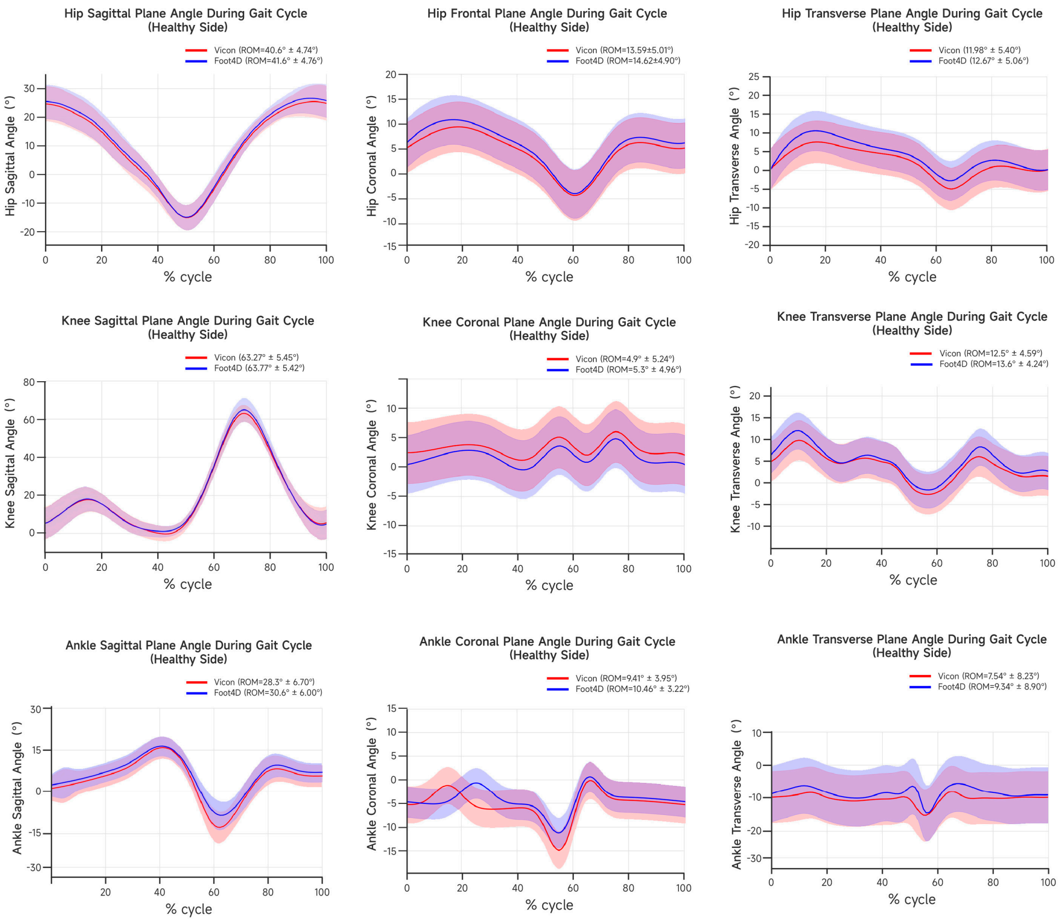

Figure S1. Comparison of three-dimensional lower limb joint kinematics between the Foot4D and Vicon systems during the gait cycle in patients with ankle injuries (healthy side). This figure presents the ensemble average angle–time curves (0–100% normalized gait cycle) for the unaffected hip joint (first row), knee joint (second row), and ankle joint (third row) in the sagittal plane (left column), coronal plane (middle column), and transverse plane (right column). The red solid line represents measurements from the Vicon system, and the blue solid line represents measurements from the Foot4D system; shaded areas indicate ±1 standard deviation (SD) for each system. The legend indicates the range of motion (ROM) and its standard deviation for each joint in each plane.
